# Drug susceptibility to etravirine and darunavir among *Human Immunodeficiency Virus Type 1*-derived pseudoviruses in treatment-experienced patients with HIV/AIDS in South Korea

**DOI:** 10.1186/s12985-015-0283-7

**Published:** 2015-04-09

**Authors:** Oh-Kyung Kwon, Sung Soon Kim, Jee Eun Rhee, Mee-Kyung Kee, Mina Park, Hye-Ri Oh, Ju-yeon Choi

**Affiliations:** Department of Immunology and Pathology, Division of AIDS, Korea National Institute of Health, Osong, Republic of Korea

**Keywords:** Phenotypic drug susceptibility, Genotypic drug resistance, Etravirine, Darunavir, Treatment-experienced, Korea

## Abstract

**Background:**

In South Korea, about 20 types of antiretroviral drugs are used in the treatment of patients with human immunodeficiency virus/acquired immune deficiency syndrome. Since 2010, raltegravir, etravirine, and darunavir have been spotlighted as new drugs for highly active antiretroviral therapy (HAART)-experienced adults with resistant HIV-1 in South Korea. In this study, we investigated potential susceptibility of pseudoviruses derived from treatment-experienced Korean patients to etravirine vs efavirenz and to darunavir vs amprenavir and indinavir using a modified single-round assay.

**Methods:**

Pseudoviruses derived from nine treatment-experienced patients infected with HIV-1 were investigated by comparison with the wild-type strain pNL4-3. The 50% inhibitory concentration (IC_50_) values were calculated and drug susceptibility was compared. The intensity of genotypic drug resistance was classified based on the ‘SIR’ interpretation of the Stanford data base.

**Results:**

Drug susceptibility was generally higher for etravirine and darunavir compared with efavirenz, amprenavir, and indinavir in pseudoviruses derived from treatment-experienced patients. Pseudoviruses derived from patients KRB4025 and KRB8014, who exhibited long-term use of protease inhibitors, showed an outside of tested drug concentration, especially for amprenavir and indinavir. However, they exhibited a lower fold-change in resistance to darunavir.

**Conclusions:**

Etravirine and darunavir have been used in HAART since 2010 in South Korea. Therefore, these antiretroviral drugs together with other newly introduced antiretroviral drugs are interesting for the optimal treatment of patients with treatment failure. This study may help to find a more effective HAART in the case of HIV-1 infected patients that have difficulty being treated.

## Background

More than 30 commercial antiretroviral drugs have been used worldwide for the treatment of patients infected with human immunodeficiency virus who show acquired immunodeficiency syndrome (HIV/AIDS). Although both mortality and morbidity have declined after the use of combinations of more than 30 commercial antiretroviral drugs [1,2], many drug-resistant variants have been reported after the initiation of highly active antiretroviral therapy (HAART). In South Korea, about 20 of the antiretroviral drugs approved by the United States Food and Drug Administration (FDA) have been administered to patients with HIV/AIDS since zidovudine was first introduced in 1991 [3]. HAART including protease inhibitors has been used in the treatment of such patients since 1997 [4]. Generally, combination therapy consists in the use of two nucleoside reverse transcriptase inhibitors (NRTIs) or of a protease inhibitor (PI) and a non-nucleoside reverse transcriptase inhibitor (NNRTI) concomitantly for the treatment of HIV/AIDS patients. However, these drugs have some problems that stem from their relatively low genetic barrier to resistance, their pill burden, the cross-resistance between them, and tolerability problems in first-generation drugs and first-line antiretroviral therapy [5,6]. Recently, etravirine was spotlighted as a second-generation NNRTI that exhibits a higher genetic barrier to resistance [7]. Etravirine was approved by the FDA in January 2008 and is reportedly effective in treatment-experienced patients infected with HIV/AIDS, as described by several research groups [8,9]. Darunavir, which was approved in June 2006, is the latest licensed protease inhibitor with in vitro activity against both wild-type (WT) and PI-resistant HIV-1 isolates, and ritonavir-boosted darunanvir exhibits clinical efficacy in patients in whom multiple PI-containing regimens have failed [10]. It has been suggested that darunavir has virological response that is superior to that achieved using comparable PIs in patients harboring PI-resistant virus [11].

Especially, etravirine and darunavir have been introduced in South Korea since 2010, although other antiretroviral drugs of the same class have been already used in the scope of HAART. To estimate drug susceptibility and fold changes in the resistance to newly introduced antiretroviral drugs, such as etravirine and darunavir, compared with generally used antiretroviral drugs of the same class, we investigated HIV-1 pseudoviruses derived from treatment-experienced patients using an established and modified phenotypic drug susceptibility assay. We analyzed genetic variations in the HIV-1 *gag-pol* sequences to investigate actual phenotypic drug resistance interpretation in vitro in contrast to predicted genotypic drug resistance interpretation based on the Stanford HIV Drug Resistance Database (Stanford DB), focusing specifically on NNRTI- and PI-related drug resistance.

## Results

### The characteristics of HIV-1 derived from treatment-experienced patients

Table 1 shows drug resistance-related mutations and amino acid polymorphisms for *pol* in patients with nine treatment experience who were infected with HIV subtype B. All treatment-experienced patient-derived pseudoviruses, with the exception of those derived from patient KRC0064, were predicted to be resistant to more than one NRTI, as assessed by genotyping. The analysis of genotypic drug resistance in all patients, with the exception of patient KRC0064, suggested that it was resistant to at least one class of antiretroviral drugs (Table 1). Most of the patients were treated with HAART combining NRTI and PI or NRTI and NNRTI. The drug susceptibility based on the IC_50_ value and fold change (FC) was calculated relative to that of the WT (Table 1).

**Table 1:** **Comparison of drug resistance level between genotype and phenotype, focusing on the HIV-1** ***gag-pol*** **region**

**Strain or isolates** ^**§**^	**Characteristics (PI/NRTI/NNRTI-drug resistance-related mutation sites)**	**Subtype**		**Antiretroviral drugs**				
	**Sample date**	**(PR/RT)**		**APV**	**IDV**	**DRV**	**EFV**	**ETR**
HIV-1 pNL4-3	Wild type (None/None/None)	B/B	Genotype^**a**^ (Mutation Score)	S (0)	S (0)	S (0)	S (0)	S (0)
			Phenotype^**b**^ (IC_50_, FC*)	7.17 nM, 1	13.5 nM, 1	6.80E–01 nM, 1	4.40E–02 nM, 1	1.34E–02 nM, 1
KRB8067	Treatment-experienced patient infected with HIV. Treatment with AZT, 3TC, and IDV from Jul. 2003 to Apr. 2006	B/B	Genotype (Mutation Score)	R (60)	R (80)	S (0)	S (0)	S (0)
	(M46I,I54V,V82A,L10F/M184V/None) Apr. 24, 2006		Phenotype (IC_50_, FC)	3.52 nM, 0.5	34.4 nM, 2.6	8.58E–02 nM, 0.1	5.00E–02 nM, 1.1	7.99E–02 nM, 6.0
KRC2065	Treatment-experienced patient infected with HIV. Treatment with AZT, 3TC, and IDV from May 2002 to Oct. 2004.	B/B	Genotype (Mutation Score)	S (0)	S (0)	S (0)	S (0)	S (0)
	(L10V/M41L,M184V/None) Oct. 14, 2004		Phenotype (IC_50_, FC)	171 nM, 23.9	91.7 nM, 6.8	43.8 nM, 64.4	5.47 nM, 124.3	3.84E–01 nM, 28.7
KRB5018	Treatment-experienced patient infected with HIV. Treatment with AZT, ddI, 3TC, IDV, and EFV from Jul. 1998 to Dec. 2004.	B/B	Genotype (Mutation Score)	S (0)	S (0)	S (0)	R (90)	I [15]
	(L10I/M41L,L74V,M184V,L210W,T215Y,K219N/L100I,K103N) Dec. 27, 2004		Phenotype (IC_50_, FC)	44.2 nM, 6.2	4.83 nM, 0.4	2.53E–01 nM, 0.4	119 nM, 2704.6	9.20E–01 nM, 68.7
KRC2092	Treatment-experienced patient infected with HIV. Treatment with AZT, 3TC, IDV, and EFV from Jan. 2003 to Nov. 2004.	B/B	Genotype (Mutation Score)	S (0)	S (0)	S (0)	R (80)	S (5)
	(None/D67N,M184V,L210W,T215Y/A98G,K103N,K238T) Nov. 29, 2004		Phenotype (IC_50_, FC)	81.9 nM, 11.4	53.4 nM, 4.0	1.45 nM, 2.1	10.5 nM, 238.6	5.00E–02 nM, 3.7
KRB4025	Treatment-experienced patient infected with HIV. Treatment with AZT, LPV/RTV, ddI, and 3TC from May 2003 to Feb. 2009.	B/B	Genotype (Mutation Score)	R (145)	R (90)	I (30)	I (30)	I (30)
	(M46I,L76V,V82C,I84V,L10I,V11I/M41L,T69D,L210W,T215Y/Y181C) Feb. 9, 2009		Phenotype (IC_50_, FC)	<1e–004 nM, >>>	>1000 nM, >>	164 nM, 241.2	7.51 nM, 170.7	5.77 nM, 430.6
KRB8014	Treatment-experienced patient infected with HIV. Treatment with AZT, IDV, 3TC, ddI, EFV, and LPV/RTV from Jun. 1998 to Mar. 2009.	D/B	Genotype (Mutation Score)	R (140)	R (150)	I (20)	R (90)	I (15)
	(M46L,I54V,L76V,V82A,L90M,L10V,K43T,A71V/M41L,L74V,M184V,L210W,T215C,K219E/L100I,K103N) Mar. 5, 2009		Phenotype (IC_50_, FC)	<1e–004 nM, >>>	>1000 nM, >>	162 nM, 238.2	6.34E–02 nM, 1.4	6.03E–02 nM, 4.5
KRC3221	Treatment-experienced patient infected with HIV.	B/B	Genotype (Mutation Score)	S (10)	I (30)	S (0)	I (15)	S (0)
	(V82A,L10I/L210W,T215Y/K103T) Nov. 1, 2008		Phenotype (IC_50_, FC)	141 nM, 19.7	324 nM, 24.0	1.70 nM, 2.5	8.41E–01 nM, 19.1	3.04E–02 nM, 2.3
KRC0064	Treatment-experienced patient infected with HIV. Treatment with AZT, 3TC, and LPV/RTV from Feb. 2009 to Oct. 2009.	B/B	Genotype (Mutation Score)	S (0)	S (0)	S (0)	S (0)	S (0)
	(None/None/None) Oct. 22, 2009		Phenotype (IC_50_, FC)	1.69 nM, 0.2	8.98E–01 nM, 0.1	5.87E–02 nM, 0.1	4.10E–01 nM,9.3	8.83E–02 nM,6.6
KRC4543	Treatment-experienced patient infected with HIV. Treatment with 3TC, EFV, and AZT from May 2008 to Jan. 2009	B/B	Genotype (Mutation Score)	I (20)	I (20)	S (0)	R (90)	I (30)
	(L90M,L10I,A71V/M41L,T69i,M184V,T215F/K101P,K103N)		Phenotype (IC_50_, FC)	21.5 nM, 3.0	129 nM, 9.6	2.26 nM, 3.3	1.39E–01 nM, 3.2	7.73E–02 nM, 5.8
	Jan. 8, 2009							

^a^Genotype means predicted genotypic drug resistance against antiretroviral drugs based on the Stanford DB. The Mutation Score is provided as the sum of the scores of each drug-resistance-related mutation site.

Key: S, susceptible (susceptible, potential low-level resistance); I, intermediate (low-level, intermediate-level resistance); R, resistant (high-level resistance).

(ARV Resistance Estimates based on the Stanford DB were evaluated as overall scores of fold increase in resistance as ‘Susceptible’ (0–14), ‘Intermediate’ (15–59), and ‘Resistant’ (>60) compared with the WT-derived pseudovirus as a standard).

PR means protease and RT means reverse transcriptase.

Three protease inhibitors (PIs): indinavir sulfate (IDV, Merck & Co., Inc., Whitehouse Station, NJ, USA), amprenavir (APV, GlaxoSmithKline (GSK)), and darunavir (DRV, Tibotec).

Two non-nucleoside reverse transcriptase inhibitors (NNRTIs): efavirenz (EFV, Merck) and etravirine (ETR, Tibotec).

^§^All patient-related data were anonymized before analysis and all indications were converted into designated labels at the Korea Centers for Disease Control & Prevention (KCDC).

^b^Phenotype means phenotypic drug susceptibility based on 50% inhibitory concentration and calculated fold change.

*FC (fold change) in ‘Phenotype’ means fold resistance values compared with the WT pseudovirus based on data obtained using a modified phenotypic drug susceptibility assay. The drug resistance index calculated by phenotypic drug susceptibility was compared with that of WT-derived pseudovirus by fold change.

### IC_50_ determination for two NNRTIs and three PIs using WT-derived pseudoviruses

**Figure 1:**
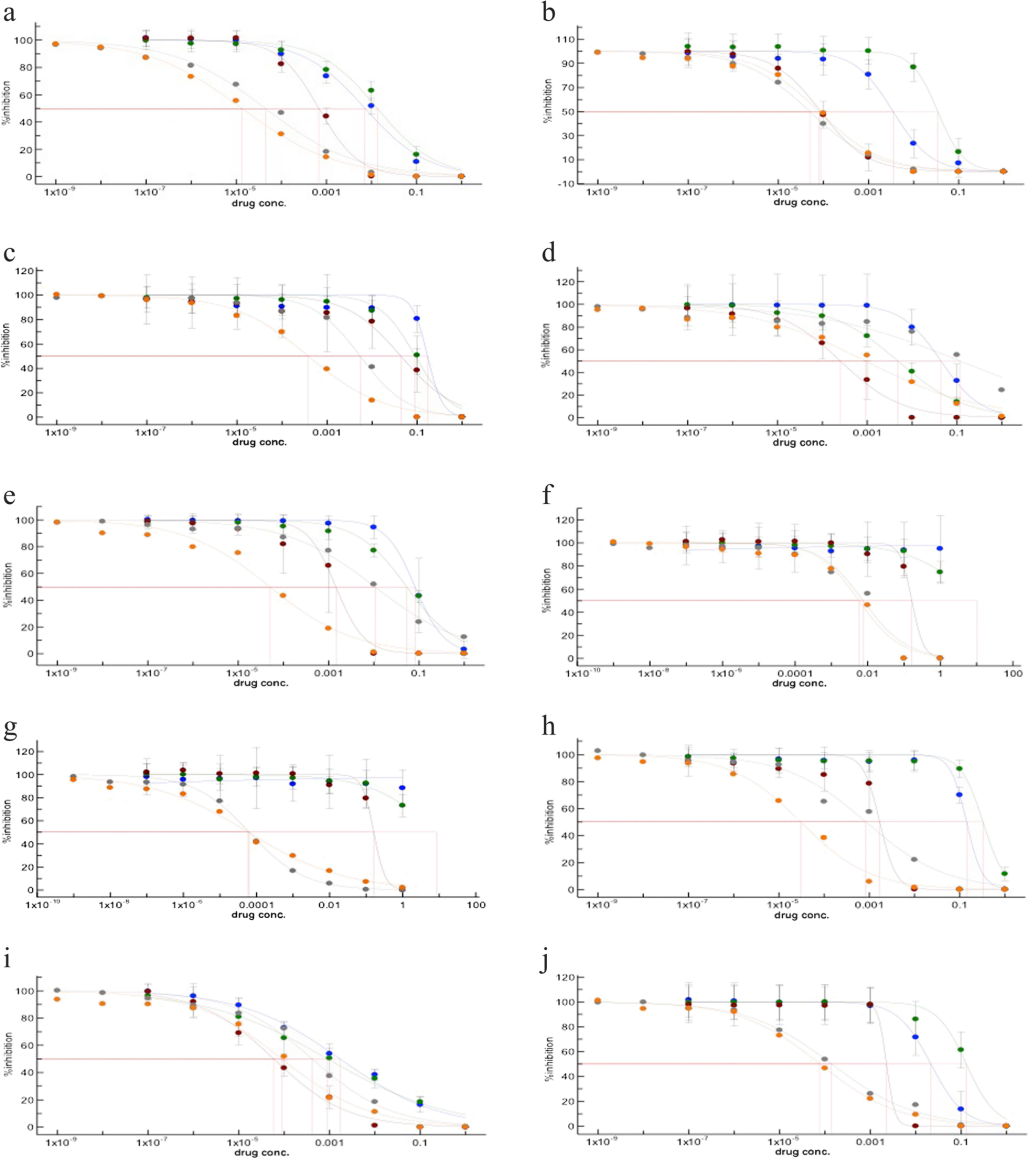
**Comparison of IC**_**50**_ **against three PIs (amprenavir, indinavir, and darunavir) and two NNRTIs (etravirine and efavirenz) for nine treatment-experienced patient-derived recombinant pseudoviruses based on simultaneous measurement of drug susceptibility.** Each color indicates the following antiretroviral drugs: blue (amprenavir), green (indinavir), brown (darunavir), gray (efavirenz), and orange (etravirine). Each experiment was repeated three times. **(a)** pNL4-3, WT; **(b)** KRB8067; **(c)** KRC2065; **(d)** KRB5018; **(e)** KRC2092; **(f)** KRB4025; **(g)** KRB8014; **(h)** KRC3221; **(i)** KRC0064; **(j)** KRC4543.

The IC_50_ for five antiretroviral drugs was analyzed using WT-derived pseudoviruses to establish a standard level using XLfit (IDBS). The following antiretroviral drugs were tested in parallel: efavirenz and etravirine, amprenavir, indinavir, and darunavir. Figure 1 shows a sigmoidal dose–response curve between serially diluted drugs and infectivity. For each antiretroviral drug, the IC_50_ was as follows (in descending order for each drug category): efavirenz (4.40E–02 nM) and etravirine (1.34E–02 nM) among NNRTIs; and indinavir (13.5 nM), amprenavir (7.17 nM), and darunavir (6.80E–01 nM) among PIs. The FC in drug resistance was compared with the IC_50_ value of the WT against each antiretroviral drug (Figure 1).

### Fold resistance to etravirine and darunavir compared with generally used drugs of the same class

Some pseudoviruses derived from treatment-experienced patients showed a more than 100-fold higher level of phenotypic drug resistance than did the WT, based on FC. However, the drug susceptibility to new antiretroviral drugs was higher than that observed for the generally used antiretroviral drugs in these cases (Table 1 and Figure 1). In the case of etravirine, the overall IC_50_ values were generally lower than those of darunavir. In addition, most patient-derived pseudoviruses showed higher drug susceptibility to etravirine compared with efavirenz (Table 1 and Figure 1). Therefore, the resistance of pseudoviruses derived from treatment-experienced patients to etravirine was generally lower than that observed for efavirenz, with the exception of those derived from patient KRB8067. In particular, patient KRC4543, who was infected with the 69 insertion complex in reverse transcriptase, exhibited a 3- and 6-FC in resistance to efavirenz (0.139 nM) and etravirine (0.0773 nM) compared with WT, respectively.

The treatment-experienced patients who received a PI-containing regimen over 6 years (KRB8014 and KRB4025) showed the highest level of FC in the resistance to amprenavir, indinavir, and darunavir. Pseudoviruses derived from patients KRB8014 and KRB4025 showed a more than 200-FC in the resistance to darunavir. Conversely, the IC_50_ value against APV and IDV was not calculated in these patients because the pseudoviruses derived from them had a level of phenotypic drug resistance that was too high (Table 1 and Figure 1).

### Comparison of the results of phenotypic and genotypic drug resistance assays

Generally, there was some discordance between genotypic and phenotypic drug resistance in this study. Phenotypic drug susceptibility according to FC was generally higher for etravirine and darunavir compared with efavirenz, amprenavir, and indinavir for each pseudovirus derived from treatment-experienced patients infected with HIV-1 (Table 1 and Figure 1). Darunavir-related resistance showed the most similar pattern between genotype and phenotype. Other antiretroviral drugs, such as amprenavir, indinavir, efavirenz, and etravirine, exhibited somewhat different patterns between genotype and phenotype.

In the case of amprenavir and indinavir in patient KRB8067, the phenotypic drug susceptibility was a little higher and similar to that of the WT, compared with the predicted genotypic drug resistance, with a mutation score of 60 and 80, respectively (Table 1).

In patients KRB8014 and KRC4543, the genotypic drug resistance-related NNRTIs suggested that they were ‘resistant’ for efavirenz and an ‘intermediate’ status for etravirine, whereas the phenotypic drug susceptibility was similar to that of the WT and less than 6 FC. In the case of patient KRB5018, both results show similar pattern that efavirenz and etravirine related genotypic drug resistance was predicted to be ‘resistant’ (mutation score 90), ‘intermediate’ (mutation score 15), and phenotypic drug resistance was also 2704.6 FC, 68.7 FC, respectively. The etravirine-related FC was highest in patient KRB4025 (430.6 FC) (Table 1).

In patient KRC0064, drug resistance was predicted to be susceptible for all antiretroviral drugs by genotyping, although this patient had been receiving a regimen of zidovudine, lamivudine, and lopinavir/ritonavir for more than 8 months. The drug susceptibility for PIs was higher than that of the WT, and that of NNRTIs was a little higher than that of the WT. These predicted results were similar between genotypic and phenotypic drug resistance.

KRC4543, a patient who was infected with the 69 insertion complex in reverse transcriptase, exhibited a lower phenotypic drug resistance compared with the predicted genotypic drug resistance. The genotypic drug resistance was suggested that they were ‘intermediate’ (mutation score of 20), whereas the phenotypic drug susceptibility in resistance to amprenavir was lower (3.0 FC). Moreover, values of 9.6 FC for indinavir and 3.3 FC for darunavir were observed in patient KRC4543. In contrast, phenotypic drug susceptibility was higher for both etravirine (5.8 FC) and efavirenz (3.2 FC), whereas genotypic drug resistance was predicted as being ‘intermediate’ (mutation score of 30 because of the K101P and K103N mutations) and ‘resistant’ (mutation score of 90 because of the K101P and K103N mutations), respectively.

## Discussion

The genotypic drug resistance, HIV viral load and CD4 T cell counts are generally used to providing additional information to permit optimal treatments for patients with HIV/AIDS. Apart from drug resistance, there are limits to interpretation using such tests in terms of drug adherence and medical opinion, especially in cases of treatment failure or discordant patients with HIV/AIDS.

This study was designed to compare and estimate genotypic drug resistance and phenotypic drug susceptibility focusing on newly introduced antiretroviral drugs such as darunavir and etravirine. And, we assume that antiretroviral drugs such as darunavir and etravirine have the potential to be more efficacious in HAART-experienced patients after their introduction in 2010 in South Korea. Prior to the introduction of darunavir and etravirine, tested samples were selected and analyzed.

Etravirine was the first NNRTI to show antiviral activity in treatment-experienced adult patients with HIV-1 strains that were resistant to NNRTIs and other antiretroviral agents [12]. Etravirine has been reported as being efficacious in achieving viral suppression and improving immune function in treatment-experienced HIV-infected patients since it was approved twice-daily darunavir coadministered with ritonavir for use by treatment-experienced adults in June 2006 [8,9,13]. Etravirine is also an approved NNRTI that can overcome single point mutations, such as K103N, that confer cross-resistance to both nevirapine and efavirenz [7]. In general, the high genetic barrier of etravirine requires the accumulation of more than three resistance-associated mutations to achieve diminished drug efficacy, and the relative roles of each of these mutations in the development of such resistance are unclear [8]. Darunavir has also been reported as a second-generation PI that has a virological response that is superior to that achieved by comparable PIs in patients harboring PI-resistant viruses [11,14]. Darunavir is an HIV-1 PI with a broad-spectrum in vitro amprenavir activity in both wild-type viruses and multidrug-resistant HIV-1 strains [15]. Darunavir with low-dose ritonavir has demonstrated anti-retroviral efficacy and tolerability in clinical trials in a range of HIV-1-infected patients, and is approved for use in treatment-naïve and treatment-experienced patients in several countries [15]. Moreover, in patients with resistant viruses and few remaining treatment options, the combination of raltegravir, etravirine, and darunavir with low-dose ritonavir resulted in excellent virological outcomes in the AIDS and viral hepatitis (ANRS) 139 TRIO trial [16].

Etravirine-related resistance mutation sites were reported to exhibit more complex combinations than do other NNRTIs, such as nevirapine and efavirenz [6]. Etravirine-related resistance mutations are different from those of other same class of drugs (V90I, A98G, L100I, K101E/H/P, V106I, E138A/G/K, V179D/F/T, Y181C/I/V, G190S/A, and M230L), although only one or two mutation sites, such as K103N and Y181C, affect high-level resistance in other first-generation NNRTIs [6,14]. In our study, patients KRC2092 and KRC5018 had values of 238.6 FC and 2704.6 FC for efavirenz, in contrast with 3.7 FC and 68.7 FC for etravirine compared with the WT. This suggests that etravirien is more effective than efavirenz in these treatment-experienced patients. However, the predicted genotypic drug resistance was similar, with an intermediate status (mutation score of 30 because of the Y181C mutation), whereas the phenotypic drug susceptibility was predicted to be a little higher for efavirenz than for etravirine in patient KRB4025-derived pseudoviruses: 170.7 FC for efavirenz and 430.6 FC for etravirine. Xu et al. reported that the addition of E138K to Y181C also decreased the level of resistance to etravirine compared with that observed for Y181C [17]. However, Y181C was reported in only one case (KRB4025), and no patient in our study group carried the E138K mutation.

In fact, the results of the phenotypic assay may provide incomplete information, such as FC in IC_50_. Nevertheless, the phenotypic assay can be helpful in promoting better medical treatment by providing information such as the interpretation of mutual connections and cross-resistance among drug-resistant HIV variants. Therefore, the application and accumulation of data from phenotypic drug susceptibility assays have the potential of providing helpful information for predicting drug resistance.

## Conclusion

The application of this modified phenotypic drug susceptibility study is expected to help predict drug resistance as a guideline for clinicians to obtain a combined interpretation among genotypic resistance, phenotypic susceptibility, and effective clinical treatments before the introduction of modified HAART regimens. Therefore, we continuously need to predict the effectiveness of domestic newly introduced antiretroviral drugs in a large antiretroviral drug-based HAART-experienced patient groups.

## Methods

### Amplification and Insertion of patient-derived HIV-1 *gag-pol* gene into pNL4-3-ΔE-GFP

Purified *gag-pol* PCR products derived from patients were cloned into pNL4-3-ΔE-GFP (green fluorescent protein) by ligation to the *Apa* I/*Age* I fragment of pNL4-3-ΔE-GFP (NIH AIDS Research & Reference Reagent Program) [18]. Selected positive clones were kept at −80°C in 20%–25% glycerol stocks. Positive-clone-derived DNA was prepared using HiSpeed Plasmid Midi Kits (Qiagen, Hilden, Germany).

### Transfection, pseudovirus production, and quantification of infectivity

The transfection and infection processes were modified from methods described previously [19]. 293 T cells were cotransfected with wild-type (WT, pNL4-3-ΔE-GFP) or recombinant pNL4-3-ΔE-GFP and pVSV-G using Lipofectamine 2000 (Invitrogen). The pseudoviruses were obtained at 48 h posttransfection and filtered using Steriflip filters (Millipore. Madison, WI, USA).

### Phenotypic drug susceptibility assay

For measuring phenotypic drug susceptibility against antiretroviral drugs, we used five antiretroviral drugs. Each PI was used at a concentration that ranged from 1000 to 10^−3^ nM 4 h after transfection using 24-well plates. Viral infectious units were determined by counting the number of β-Gal + cell colonies using 10-fold dilutions that gave between 150 and 200 cell colonies. Each NNRTI (1000 to 10^−5^ nM) was added to the TZM-bL cell line at the start of infection. The β-galactosidase activity was measured by X-gal staining on day 2 after infection. Three tests for each drug concentration were executed, and relative infectivity was calculated by direct counting of blue foci. The 50% inhibitory concentration (IC_50_) values were calculated by curve fitting of XLfit4.2 (IDBS, Guildford, Surrey, UK). Fold changes in resistance values were compared with the WT-derived pseudovirus based on data obtained using a modified phenotypic drug susceptibility (In-house Phenotype) and the genotypic drug resistance (Stanford DB) (Table 1).

### Prediction of drug resistance level using genotypic resistance assay

The conditions of reverse transcription polymerase chain reaction (RT–PCR) and PCR were as described previously [19]. The PCR product of *gag*-*pol* (about 1.5 kb) was used for ligation after purification using NucleoFast® 96 PCR (MACHEREY-NAGEL GmbH & Co.KG). The PCR product of *pol* gene sequences was subjected to direct sequencing in an ABI Prism Dye Terminator Cycle Sequencing Ready Reaction Kit (PerkinElmer, Waltham, MA, USA) in an automated sequencer (ABI Prism 3110 DNA sequencer, Applied Biosystems, Foster City, CA, USA). The *pol* nucleotides and encoded amino acid sequences were aligned using EditSeq and MegAlign programs in the Lasergene software package (version 5.06; DNASTAR Inc., Madison, WI, USA). The interpretation of the resistant mutations was based on the Stanford DB (Drug Resistance Algorithm, Beta Test (version 6.3)), release notes for HIV seq, HIV db, HIV alg. http://sierra2.stanford.edu/sierra/servlet/JSierra?action=sequenceInput).

The institutional review board “KCDC Research Ethics Committee (no. 2012-05-11-9)” approved this study.

## References

[1] Antiretroviral drugs used in the treatment of HIV infection. US Food and Drug Administration. http://www.fda.gov/forpatients/illness/hivaids/treatment/ucm118915.htm.

[2] Tang MW, Shafer RW (2012). HIV-1 antiretroviral resistance scientific principles and clinical applications. Drugs.

[3] Sung H, Foley BT, Bae IG, Chi HS, Cho YK (2001). Phylogenetic analysis of reverse transcriptase in antiretroviral drug-naïve Korean HIV type 1 patients. AIDS Res Hum Retroviruses.

[4] Sung H, Foley BT, Ahn SH, Kim YB, Chae JD, Shin YO (2003). Natural polymorphisms of protease in protease inhibitor-naive HIV-1 infected patients in Korea: a novel L63M in subtype B. AIDS Res Hum Retroviruses.

[5] Paredes R, Clotet B (2010). Clinical management of HIV-1 resistance. Antiviral Res.

[6] Johnson VA, Calvez V, Gunthard HF, Paredes R, Pillay D, Shafer RW (2013). Update of the drug resistance mutations in HIV-1: March 2013. Top Antivir Med.

[7] Melikian GL, Rhee SY, Varghese V, Porter D, White K, Taylor J (2014). Non-nucleoside reverse transcriptase inhibitor (NNRTI) cross-resistance: implications for preclinical evaluation of novel NNRTIs and clinical genotypic resistance testing. J Antimicrob Chemother.

[8] Elsayed RK, Caldwell DJ (2010). Etravirine: a novel nonnucleoside reverse transcriptase inhibitor for managing human immunodeficiency virus infection. Am J Health Syst Pharm.

[9] Towner WJ, Cassetti I, Domingo P, Nijs S, Kakuda TN, Vingerhoets J (2010). Etravirine: clinical review of a treatment option for HIV type-1-infected patients with non-nucleoside reverse transcriptase inhibitor resistance. Antivir Ther.

[10] Sterrantino G, Zaccarelli M, Colao G, Baldanti F, Di Giambenedetto S, Carli T (2012). Genotypic resistance profiles associated with virological failure to darunavir-containing regimens: a cross-sectional analysis. Infection.

[11] Talbot A, Grant P, Taylor J, Baril JG, Liu TF, Charest H (2010). Predicting tipranavir and darunavir resistance using genotypic, phenotypic, and virtual phenotypic resistance patterns: an independent cohort analysis of clinical isolates highly resistant to all other protease inhibitors. Antimicrob Agents Chemother.

[12] de Bethune MP (2010). Non-nucleoside reverse transcriptase inhibitors (NNRTIs), their discovery, development, and use in the treatment of HIV-1 infection: a review of the last 20 years (1989–2009). Antiviral Res.

[13] Peeters M, Vingerhoets J, Tambuyzer L, Azijn H, Hill A, De Meyer S (2010). Etravirine limits the emergence of darunavir and other protease inhibitor resistance-associated mutations in the DUET trials. AIDS.

[14] Wensing AM, van Maarseveen NM, Nijhuis M (2010). Fifteen years of HIV protease inhibitors: raising the barrier to resistance. Antiviral Res.

[15] Dierynck I, De Meyer S, Lathouwers E, Vanden Abeele C, Van De Casteele T, Spinosa-Guzman S (2010). In vitro susceptibility and virological outcome to darunavir and lopinavir are independent of HIV type-1 subtype in treatment-naïve patients. Antivir Ther.

[16] Barrail-Tran A, Yazdanpanah Y, Goldwirt L, Chene G, Colin C, Piketty C (2010). Pharmacokinetics of etravirine, raltegravir and darunavir/ritonavir in treatment-experienced patients. AIDS.

[17] Xu HT, Oliveira M, Asahchop EL, McCallum M, Quashie PK, Han Y (2012). Molecular mechanism of antagonism between the Y181C and E138K mutations in HIV-1 reverse transcriptase. J Virol.

[18] Zhang H, Zhou Y, Alcock C, Kiefer T, Monie D, Siliciano J (2004). Novel single-cell-level phenotypic assay for residual drug susceptibility and reduced replication capacity of drug-resistant human immunodeficiency virus type 1. J Virol.

[19] Choi JY, Kwon OK, Choi SY, Park YK, Kim SS (2011). Drug susceptibility of human immunodeficiency virus type 1-derived pseudoviruses from treatment-experienced patients to protease inhibitors and reverse transcriptase inhibitors, using a modified single-round assay. J Clin Virol.

